# Case of Calculi Found, upon Dissection, in the Kidneys and Bladder of Urine of a Small Spaniel Bitch

**Published:** 1818-10

**Authors:** John Maclean

**Affiliations:** Edinburgh.


*For the London Medical and Physical Journal.*

*Case of Calculi found, upon Dissection, in the Kidneys and Bladder of Urine of a small Spaniel Bitch.* By John Maclean, M.D. Edinburgh.

A SMALL spaniel bitch, which I kept for several years, and which had always seemed to be in perfect health, was observed for some time before its death, though previously remarkably cleanly, to void its urine frequently in small quantities about the house, with apparent difficulty and uneasiness, which induced a suspicion of some disease in the urinary passages.

She was at last found dead in her bed, after having been for several months in her usual health, and betraying no particular uneasiness.

On examining the body after death, a sandy concretion was found in each kidney; they were rough, not very hard, of an oval form, and about the size of a small hen's egg. The bladder was completely occupied by seven very smooth and hard calculi, with flattened faces adapted to each other, and resembling polished pebbles, having evidently been the gradual product of the gravelly contents of the kidneys. The latter, being immersed in strong vinegar, were dissolved, or rather reduced to a soft pulp in a few weeks.

The calculi from the bladder were presented to the late Dr. Monro, and are, I believe, still preserved in the Anatomical Museum of the University. It was the doctor's opinion, after minutely examining them, that they would have resisted the action of the strongest acid, and that, consequently, such cases could only be relieved by lithotomy, although their rudiments in the kidneys might, as is well known, by the use of proper medicines, have been dissolved and evacuated with the urine.

*August* 18, 1818.

---

